# Transduction of skin-migrating dendritic cells by human adenovirus 5 occurs via an actin-dependent phagocytic pathway

**DOI:** 10.1099/jgv.0.000581

**Published:** 2016-10-13

**Authors:** Efrain Guzman, Geraldine Taylor, Jayne Hope, Rebecca Herbert, Carolina Cubillos-Zapata, Bryan Charleston

**Affiliations:** ^1^​ The Pirbright Institute, Ash Road, Woking, Surrey GU240NF, UK; ^2^​ The Roslin Institute University of Edinburgh, Easter Bush, Midlothian EH259RG, UK

**Keywords:** dendritic cells, adenovirus entry, afferent lymph dendritic cells, receptor-independent endocytosis

## Abstract

Dendritic cells (DC) are central to the initiation of immune responses, and various approaches have been used to target vaccines to DC in order to improve immunogenicity. Cannulation of lymphatic vessels allows for the collection of DC that migrate from the skin. These migrating DC are involved in antigen uptake and presentation following vaccination. Human replication-deficient adenovirus (AdV) 5 is a promising vaccine vector for delivery of recombinant antigens. Although the mechanism of AdV attachment and penetration has been extensively studied in permissive cell lines, few studies have addressed the interaction of AdV with DC. In this study, we investigated the interaction of bovine skin-migrating DC and replication-deficient AdV-based vaccine vectors. We found that, despite lack of expression of Coxsackie B–Adenovirus Receptor and other known adenovirus receptors, AdV readily enters skin-draining DC via an actin-dependent endocytosis. Virus exit from endosomes was pH independent, and neutralizing antibodies did not prevent virus entry but did prevent virus translocation to the nucleus. We also show that combining adenovirus with adjuvant increases the absolute number of intracellular virus particles per DC but not the number of DC containing intracellular virus. This results in increased trans-gene expression and antigen presentation. We propose that, in the absence of Coxsackie B–Adenovirus Receptor and other known receptors, AdV5-based vectors enter skin-migrating DC using actin-dependent endocytosis which occurs in skin-migrating DC, and its relevance to vaccination strategies and vaccine vector targeting is discussed.

## Introduction

Vaccines based on replication-incompetent adenovirus (AdV) vectors are safe and highly immunogenic, capable of inducing a full spectrum of adaptive humoral and cell-mediated immune responses and of inducing protective immunity in a number of animal species including man (Dicks *et al.*, 2015; Green *et al.*, 2015; Taylor *et al.*, 2015).

Human adenovirus 5 (AdV5 and sometimes referred to as HAdV-C5), a species C adenovirus, is the most commonly studied adenovirus vector for both gene therapy and vaccination and, thus, it is also the most studied in terms of cell entry, host responses and gene expression (Smith *et al.*, 2010). Epithelial cell models have been used to describe the mechanism of AdV5 entry and trafficking to the nuclear membrane (Svensson & Persson, 1984; Wolfrum & Greber, 2013). It is generally accepted that the first step in AdV5 entry to its target cell is the binding of the virus’ fibre protein to CAR (Coxsackie B–Adenovirus Receptor), followed by the binding of the RGD motif on the penton base to cellular integrins (α_v_β_3_ and α_v_β_5_). This promotes virus endocytosis into clathrin-coated vesicles and triggers the first step of the uncoating program (Burckhardt *et al.*, 2011). Once inside the cell, the virus exits endosomal vesicles to the cytosol, where it utilizes microtubule motors to traffic to the nuclear membrane (Bremner *et al.*, 2009) and deliver its DNA through the nuclear pore (Puntener *et al.*, 2011).

Although CAR has been shown to be the primary receptor for AdV5 entry in epithelial cells, CAR is not expressed on all cells that can be infected with AdV5. For example, infection of Kupffer cells is mediated by human blood coagulation factor X binding to AdV5 hexon (Alba *et al.*, 2009; Kalyuzhniy *et al.*, 2008; Waddington *et al.*, 2008) and entry of AdV5 to human peripheral blood monocyte-derived dendritic cells (DC) involves CD209 (Adams *et al.*, 2009).

Since their identification (Steinman & Cohn, 1973), DC have become increasingly recognized for their crucial role as initiators and regulators of immune responses. Many studies of DC biology rely on the isolation of monocytes or macrophages from blood or tissues (such as spleen or bone marrow) (Rossi & Young, 2005; Steinman, 1991) followed by maturation with IL-4 and granulocyte-macrophage colony-stimulating factor or the harvest of tissues followed by isolation of resident DC. The cannulation of lymphatic vessels provides *ex vivo* DC derived from relevant anatomical sites such as the skin that drains sites of vaccination (Hemati *et al.*, 2009; Hope *et al.*, 2006; Schwartz-Cornil *et al.*, 2006). Due to the complexity of the surgical procedure to cannulate lymphatic vessels, this is most easily performed in large animals, such as cattle and sheep. We and others have described afferent lymph dendritic cells (ALDC) as being FSC^high^ MHCII^+^ DEC205^+^ CD11c^+^ CD8α^−^ (Cubillos-Zapata *et al.*, 2011; Gliddon *et al.*, 2004; Hope *et al.*, 2006). Within this population, subpopulations of DC expressing various levels of SIRPα (CD172a), CD11a, CD26 and CD13 have also been described (Brooke *et al.*, 1998; Gliddon *et al.*, 2004; Gliddon & Howard, 2002; Howard *et al.*, 1997). Of these, the SIRPα^+^ DC population is targeted by various vaccine vectors and these cells are more efficient at antigen presentation compared to SIRPα^neg/low^ ALDC (Guzman *et al.*, 2012; Hope *et al.*, 2012).

In the current study, we describe the interaction between replication-deficient AdV5 with bovine ALDC that drain the skin. We show that macropinocytosis is the principal entry mechanism for AdV5 into ALDC and that the kinetics of virus internalization are much slower than previously described for epithelial cells. We also show that virus exit from endosomal compartments does not require an acidic microenvironment. Furthermore, neutralizing antibodies do not block internalization of AdV5 but prevent trans-gene expression. Finally, we demonstrate that emulsification of AdV5 in oil-in-water adjuvants improves virus internalization into ALDC *in vitro*, increasing trans-gene expression and antigen presentation. Defining and manipulating entry pathways may enhance vaccine vector efficacy through improved antigen delivery and presentation.

## Results

### Bovine afferent lymph DC are transduced by adenovirus-based vectors despite the absence of known adenovirus receptors

**Fig. 1.**
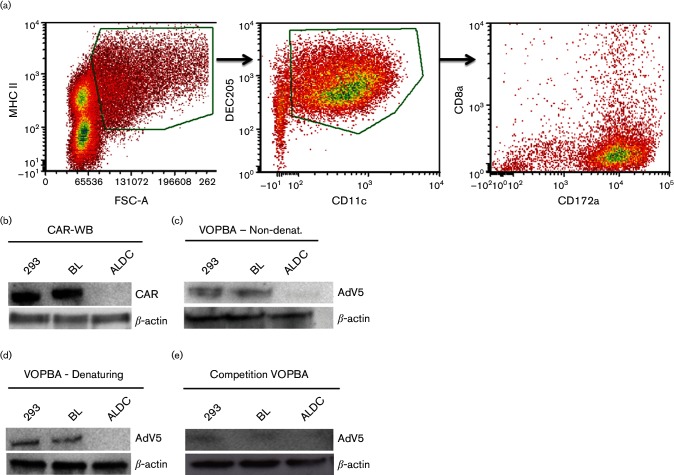
Phenotype of ALDC and expression of CAR in bovine cells. (a) The phenotype of ALDC was characterized as FSC^high^ MHCII^+^ DEC205^+^ CD11c^+^ CD8α^−,^ and 91 % of these cells were CD172a^+^. The plot is representative of all samples analysed (*n*=8 animals). (b) Expression of CAR and β-actin was assessed by Western blot on enriched membrane fractions from 293, BL or ALDC. Blot representative of three different experiments. (c and d) VOPBA was used to confirm binding of AdV5 to enriched membrane fractions from 293 and BL but not from ALDC under denaturing and non-denaturing conditions. (e) Competition VOPBA using anti-CAR antibodies to block binding of AdV5 to enriched membrane fractions from 293 and BL cells. Blots representative of five different experiments.

We have previously shown that AdV5 injected subcutaneously or intramuscularly above the site of cannulation is internalized by migrating DC between 4 and 12 h post-inoculation and that, *in vitro*, up to 40 % of ALDC can be transduced by AdV5 using a multiplicity of infection (MOI) of 100 (Cubillos-Zapata *et al.*, 2011). To define the mechanism by which AdV5 transduces ALDC (defined as FSC^high^ MHCII^+^ DEC205^+^ CD11c^+^ CD8α^−^; Fig. 1a), we initially assessed the expression of CAR on bovine cells including ALDC. CAR was detected by Western blotting in enriched membrane fractions from 293 and bovine lung (BL) cells but not membranes from bovine ALDC (Fig. 1b). We then used the virus overlay protein binding assay (VOPBA) to confirm binding of AdV5 to bovine CAR. Under denaturing and non-denaturing conditions, AdV5 bound to enriched membrane fractions from 293 and BL cells, but not from ALDC (Fig. 1c and d). To confirm that AdV5 was binding specifically to CAR, we used a rabbit polyclonal antibody raised against CAR to block binding of AdV5 to CAR in a competition VOPBA assay (Fig. 1e). These results indicate that although AdV5 can use CAR for binding to BL cells, CAR is not expressed on bovine ALDC and, therefore, AdV5 utilizes an alternative entry strategy for ALDC that is CAR independent.

**Fig. 2.**
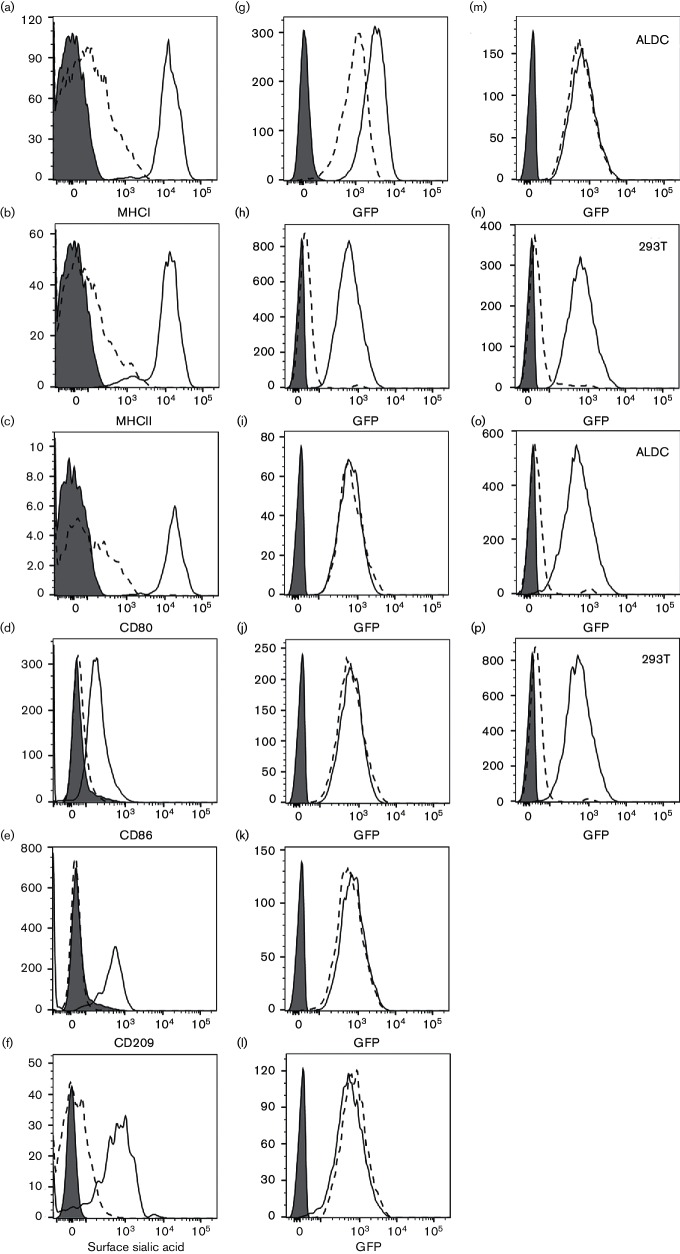
Transduction of ALDC by AdV5-GFP following removal of putative receptors from the cell surface. (a–f) Expression of putative AdV5 receptors on ALDC 24 h after treatment (dotted histograms) or mock treatment (solid histograms); grey-filled histograms: isotype controls. (g–l) Expression of GFP in AdV5-GFP-transduced untreated (solid histograms) or treated (dotted histograms) ALDC or in mock-transduced ALDC (grey-filled histograms). (m and n) Expression of GFP in AdV5-GFP-transduced ALDC and 293 cells in the presence of the RGD antagonist Cyclo(Ala-Arg-Gly-Asp-3-Aminomethylbenzoyl) (dotted histogram) or control peptide (solid histogram); grey-filled histogram: mock-transduced cells. (o and p) Expression of GFP in AdV5-GFP-transduced ALDC and 293 in the presence of AdV5 hyperimmune bovine sera (dotted histograms) or normal bovine serum (solid histograms) or mock transduced (grey-filled histograms). Plots are representative of cells from six different animals analysed in duplicate.

A number of different molecules have been implicated in AdV attachment to mononuclear phagocytic cells, including, MHC, CD80/CD86, CD209 and sialic acid (Chen & Lee, 2014). We used a combination of chymotrypsin, trypsin and papain to remove surface expression of known AdV receptors and brefeldin A to prevent their surface expression during transduction of ALDC. Following this treatment, the viability decreased to between 8 and 12 % as measured by trypan blue exclusion (data not shown). MHCI (Fig. 2a), MHCII (Fig. 2b), CD80 (Fig. 2c), CD86 (Fig. 2d) and CD209, also known as DC-SIGN (Fig. 2e), were completely removed by protease treatment and their reconstitution on the cell’s surface prevented throughout the course of the experiments. Removal of sialic acid withneuraminidase (Fig. 2f) was also complete. In all cases, these treatments did not result in a decreased transduction efficiency of ALDC by AdV5-GFP (Fig. 2g–l). Blocking the RGD-binding receptors with the antagonist Cyclo(Ala-Arg-Gly-Asp-3-Aminomethylbenzoyl) decreased the transduction efficiency of 293 cells but not ALDC (Fig. 2m and n). Finally, transduction of both 293 and ALDC was blocked by incubation in the presence of bovine hyperimmune serum against AdV5 (Fig. 2o and p). These results indicate that using the treatments indicated above, transduction of ALDC by AdV5 is not affected.

### Transduction kinetics of ALDC

**Fig. 3.**
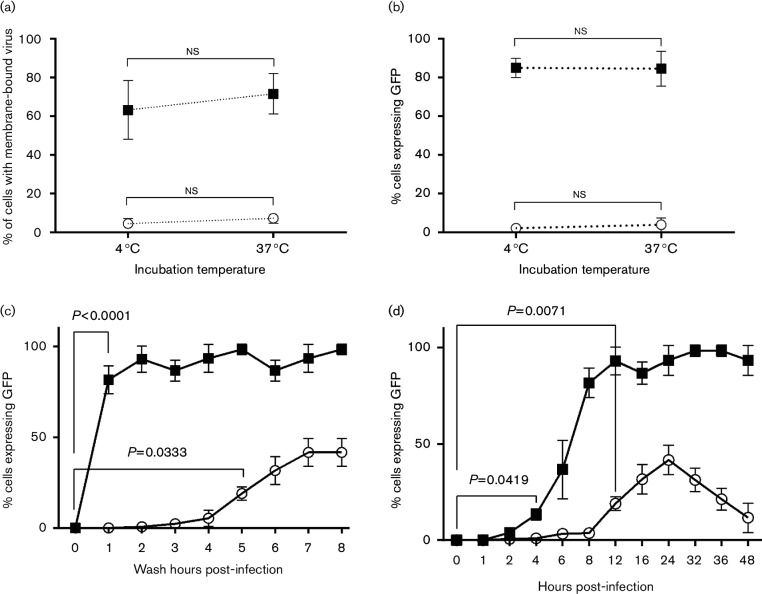
AdV5 entry and trans-gene expression in ALDC is significantly different from that in CAR-expressing 293 cells. ALDC (white circles) and 293 cells (black squares) were cultured on ice (4 °C) or at 37 °C with biotinylated AdV5-GFP for 90 min followed by washing off the inoculum. (a) Biotinylated membrane-bound virus was detected by ELISA using streptavidin-HRP. (b) Cells were transduced as before and cultured for 24 h at 37 °C; expression of the trans-gene (GFP) was detected by flow cytometry. (c) ALDC and 293 cells were cultured at 37 °C in the presence of AdV5-GFP. At 1 h intervals, aliquots were washed with PBS and the cells were allowed to recover overnight after which GFP expression was measured by flow cytometry. (d) ALDC and 293 cells were cultured at 37 °C in the presence of AdV5-GFP and without washing; GFP expression was measured by flow cytometry at 1 h intervals. Each point represents the mean of cells from six different animals tested in duplicate and error bars indicate standard error of the mean.

To investigate the transduction kinetics of ALDC by AdV5, we utilized biotinylated AdV5-GFP (MOI=100 virus particles per cell) to infect freshly isolated ALDC and 293 cells. After 90 min incubation at 4 °C or 37 °C, more than 60 % of the biotinylated AdV5-GFP had attached to 293 cells (Fig. 3a). In contrast, less than 5 % of the biotinylated AdV5-GFP had attached to ALDC at either temperature. We also blocked attachment of AdV5 to 293 cells at both temperatures using anti-CAR antibodies and, as expected, we were able to block 95–98 % of the AdV5-bio signal (data not shown). To confirm that biotinylation of the virus did not interfere with its ability to transduce cells and express encoded proteins, we also measured GFP expression in these cells by flow cytometry (Fig. 3b). A 90 min incubation of 293 cells with biotinylated AdV5 at +4 °C or 37 °C, followed by washing off the excess inoculum and subsequent overnight incubation, was sufficient to transduce more than 80 % of the cells. In contrast, <1 % of ALDC expressed GFP following the same protocol. To determine the minimum time required for transduction of cells by AdV5, we incubated 293 cells and ALDC with AdV5-GFP at 37 °C, we washed off the inoculum at 1 h intervals and we incubated the cells overnight. Transduction was measured by assessing the percentage of cells expressing GFP by flow cytometry. We determined that 1 h incubation was sufficient for AdV5 to transduce and express GFP in 293 cells but that at least 5 h was required for ALDC to be significantly transduced by the virus (Fig. 3c). To determine the kinetics of trans-gene expression, we cultured ALDC cells with AdV5-GFP (MOI=100) at 37 °C without washing, and we measured GFP expression at various time points by flow cytometry (Fig. 3d). GFP-positive 293 cells were evident at 4 h post-transduction and, by 8 h, most cells were expressing the trans-gene. In contrast, at least a 12 h incubation period was required before a significant number of ALDC expressed GFP (Fig. 3d, *P*=0.0071 % GFP expression in ALDC at 12 h compared to GFP expression at time 0). Expression of GFP in ALDC peaked after 24 h in culture. Interestingly, trans-gene expression in ALDC subsequently decreased and approached baseline levels by 48 h (Fig. 3d). These results indicate that the mechanism of AdV5 entry and trans-gene expression in ALDC is significantly different from that in CAR-expressing 293 cells.

### ALDC actively uptake adenovirus

**Fig. 4.**
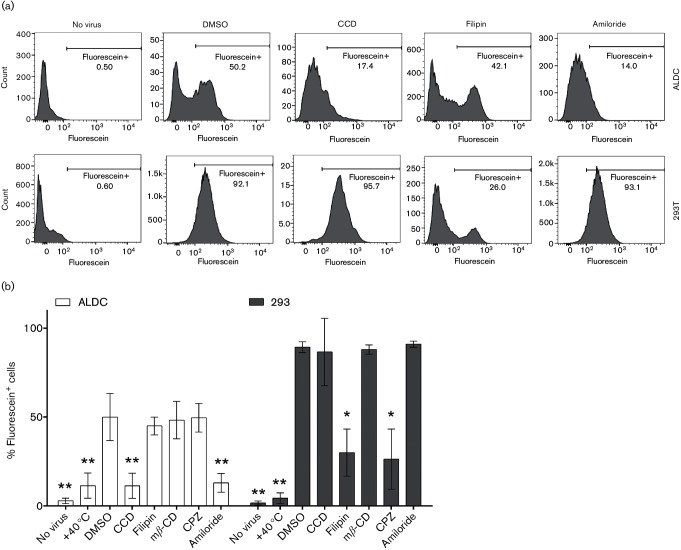
Uptake of AdV in DC is blocked by inhibitors of actin-dependent endocytosis. ALDC and 293 cells were cultured with AdV5-Fluo for 1 h at 37 °C in the presence of various endocytosis inhibitors as described in Methods. Extracellular fluorescein was then quenched with trypan blue and fluorescence was measured by flow cytometry. (a) Representative histograms showing fluorescence after various treatments. The markers indicate percentage of fluorescein-positive cells above background. (b) Bar graphs showing means and standard deviations of the percentage of fluorescein^+^ ALDC obtained from six different animals and tested in duplicate or 293 cells tested in triplicate. Asterisks indicate *P<0.05 and **P<0.005 compared to treatment with DMSO.

We then analysed the ability of a number of endocytosis inhibitors to block AdV5 entry and subsequent transduction. A fluorometric assay based on the capacity of trypan blue to quench extracellular but not intracellular fluorescein (Wan *et al.*, 1993) was modified to determine the effect of various biochemical inhibitors on the capacity for ALDC to internalize fluorescein-labelled AdV5 (AdV5-Fluo). Incubation at 4 °C for 60 min followed by quenching with trypan blue blocked internalization of AdV5-Fluo, and no fluorescence was detected in either cell type (Fig. 4b). All other treatments were carried out as described in Methods. In comparison to DMSO, treatment with the various inhibitors resulted in the following: treatment with filipin, which blocks caveolae- and cholesterol-dependent endocytosis, and chlorpromazine, which inhibits clathrin-dependent endocytosis, reduced AdV5-Fluo uptake by 293 cells, as expected, but not by ALDC. In contrast, treatment with both cytochalasin D, which blocks actin polymerization, and amiloride, which blocks Na^+^ channels, reduced the uptake of virus by ALDC (Fig. 4a and b). Treatment with methyl-β-cyclodextrin, which blocks cholesterol-dependent phagocytosis, did not have an effect on virus uptake in either cell type. Treatment with endocytosis inhibitors at the concentrations observed did not significantly increase the number of dead cells during the course of the assay (data not shown). These data indicate that actin polymerization and Na^+^/H^+^ exchange are required for AdV5 uptake by ALDC.

### Transient association of hAdV5 with early endosome markers

**Fig. 5.**
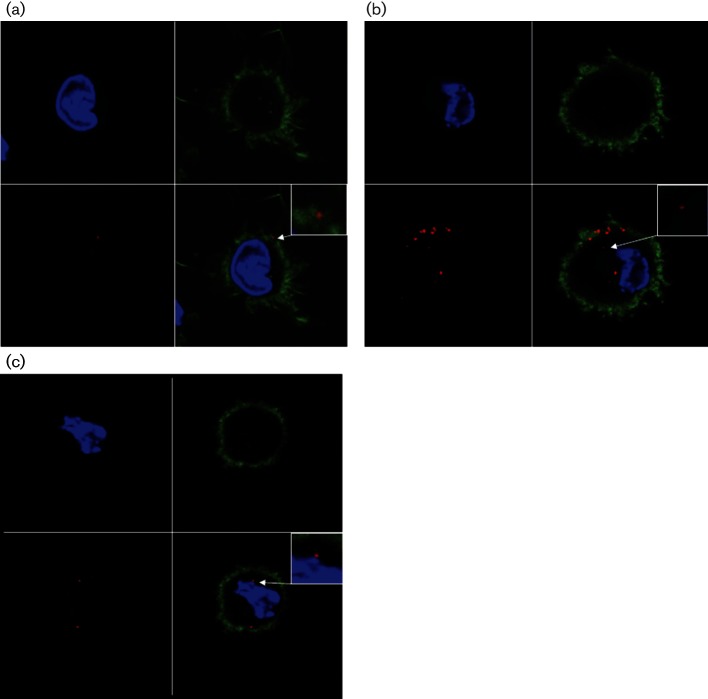
Entry of AdV5 into ALDC. ALDC were cultured at 37 °C in the presence of AF568-labelled AdV5 (MOI=100). Cells were fixed and analysed by confocal microscopy at 3 h (a), 4 h (b) or 5 h (c) post-infection. Blue: DAPI; green: Phalloidin-AF488 (for F-actin); red: AdV5-AF568. Representative samples of cells from five different animals. The scale bar represents 20 µm.

**Fig. 6.**
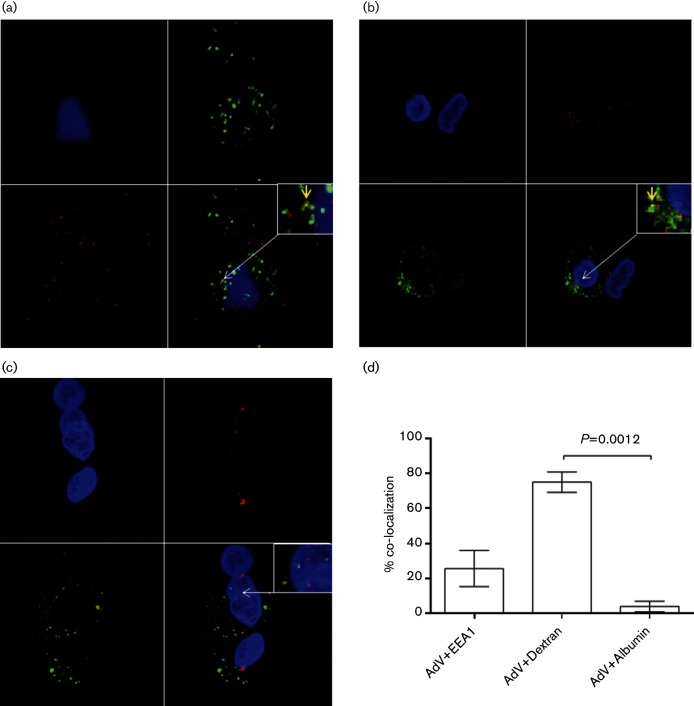
The majority of AdV are not associated with early endosomes. ALDC were cultured with AdV5-AF568 (red) and dextran-AF488 (green) or albumin-AF488 (green) for 4 h and fixed. The early endosome marker (EEA1) was detected using monospecific antibodies and AF488 secondary antibodies (green) as described in Methods, and the samples were analysed by confocal microscopy. (a) Co-localization of AdV5 and EEA1; (b) AdV5 and dextran; (c) AdV5 and albumin. Blue: DAPI; yellow: co-localization. Insets show a higher magnification of the region of interest. Arrows indicate co-localization events. Representative samples cells from four different animals. (d) Bar graph showing mean percentage of co-localization voxels from single-slice histograms across the Z-plane from at least 10 confocal images processed from cells from four different animals. Error bars indicate standard error of the mean.

Following clathrin-mediated endocytosis of AdV5 in 293 cells, virus can be detected within clathrin-coated vesicles (Ashbourne Excoffon *et al.*, 2003). These early endosomes are characterized by the presence of Rab5 and EEA1 (Christoforidis *et al.*, 1999). To determine whether entry of AdV5 into ALDC was associated with early endosomes, we cultured purified ALDC with AF568-labelled AdV5 for 1–6 h and analysed them by confocal microscopy. AdV5 could not be detected on the surface of ALDC until 3 h after addition of virus, when it was found to be associated with dendrites or cell membranes (Fig. 5a). An hour later, a greater number of virions were associated with the dendrites/cell membrane and virions were also observed within the cytoplasm (Fig. 5b). By 5 h post-infection, a proportion of virions were localized in proximity to the nucleus (Fig. 5c). We then stained AdV5-infected ALDC with EEA1-specific antibodies to determine localization of AdV5 with early endosomes. Although co-localization of AdV5-AF568 with EEA1 was observed at 4 h post-infection (Fig. 6a), co-localization events were rare, suggesting that virus exit from the phagosome/endosome occurs quickly after entry or that the majority of AdV are not associated with early endosomes. We then tracked the localization of AdV5-AF568 within ALDC using two different AF488-labelled tracers: dextran is used to track fluid-phase pinocytosis and albumin is known to enter cells using a mannose receptor endocytosis pathway. We observed co-localization of AdV568 with dextran-AF488 but not with albumin-AF488 (Fig. 6c and d), suggesting that AdV5 entry into ALDC is via fluid-phase macropinocytosis.

### Exit of AdV5 into the cytosol of ALDC is not pH mediated

**Fig. 7.**
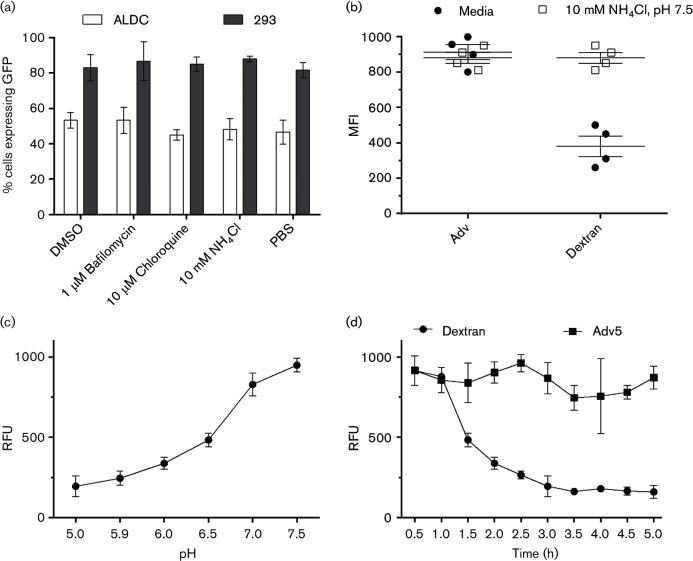
Acidification of endosomes is not required for transduction of ALDC by AdV5. (a) ALDC or 293 cells were incubated with AdV5-GFP for 24 h in the presence of various inhibitors of intracellular acidic microenvironment or with the diluents DMSO or PBS. Trans-gene expression (GFP) was measured by flow cytometry. (b) ALDC were cultured at 37 °C with fluorescein-labelled AdV5 or dextran for 4 h in the presence of normal tissue culture media or media containing 10 mM NH_4_Cl, pH 7.5. After washing fluorescein, we measured MFI by flow cytometry. (c) Relative fluorescence standard curve of fluorescein–dextran at varying pH. (d) ALDC were cultured in the presence of fluorescein-labelled AdV5 or dextran. Relative fluorescence (RFU) was measured by real-time fluorometry. Each point represents the mean of cells from four different animals tested in duplicate and error bars indicate standard error of the mean.

Following uptake, a key step in AdV5 infection is the exit of virus or virus aggregates from phagosomes/endosomes into the cytoplasm (Meier *et al.*, 2005). To define the mechanisms associated with AdV5 exit in ALDC, we treated cells with inhibitors of the intracellular acidic microenvironment such as bafilomycin, NH_4_Cl or chloroquine, 60 min prior to the addition of AdV5-GFP. Cells were then cultured for 4 h and GFP expression was measured by flow cytometry. None of the treatments significantly reduced the proportion of ALDC or 293 cells expressing GFP (Fig. 7a). To confirm the presence of acidic endosomes within ALDC, we utilized fluorescein-labelled dextran and fluorescein-labelled AdV5. The fluorescence of fluorescein is optimal at pH 7.5 and rapidly decreases under acidic conditions. Under normal culture conditions, the mean fluorescence intensity (MFI) of fluorescein-labelled dextran within ALDC decreases after 4 h incubation (Fig. 7b), confirming the presence of dextran within endosomes and their subsequent acidification. In contrast, fluorescein-labelled AdV5 within ALDC did not show a decrease in fluorescence after 4 h incubation (Fig. 7b). As expected, normalization of intracellular pH with 10 mM NH_4_Cl, pH 7.5, inhibited the reduction of fluorescence in dextran-loaded ALDC. To confirm these results, we measured the relative fluorescence of fluorescein at varying pH and generated a standard curve (Fig. 7c). ALDC were incubated with fluorescein-labelled AdV5 or dextran and fluorescence was measured by real-time fluorometry. Fig. 7(d) shows that the fluorescence of dextran in ALDC decreases over time as the fluorescein becomes protonated, but the fluorescence of AdV5 remains the same suggesting that acidic endosomes are not involved in adenovirus uncoating in ALDC.

### Neutralizing antibodies do not prevent virus entry into ALDC

An important hurdle to successful vaccination with viral vectors is the presence of neutralizing antibodies that can limit the efficacy of the vaccine. Most neutralizing antibodies are raised against the fibre protein of AdV and thus block virus attachment to its receptor (normally CAR). In light of our results, we sought to investigate the effect of neutralizing antibodies on AdV5 entry into ALDC. We incubated AdV5-568 with normal bovine sera, hyperimmune bovine sera to AdV5 or mouse monoclonal anti-hexon antibodies. Virus–antibody complexes were then added to cultures of ALDC and virus entry was assessed by confocal microscopy, 4–6 h post-infection. Interestingly, the mean number of intracellular AdV5 was significantly higher (*P*=0.0027) in the presence of bovine hyperimmune sera or mouse anti-hexon antibodies compared to normal bovine sera (Fig. S1a, available in the online Supplementary Material). However, even after 6 h in culture, virus complexed with antibody did not migrate to the nuclear membrane but it remained in the mid-cytoplasm (Fig. S1b) whereas AdV5, incubated with normal bovine sera, migrated and was located proximal to the nuclear membrane as expected (Fig. S1c). These data show that the presence of neutralizing antibodies does not prevent attachment of the virus to the cell but rather blocks a process downstream of virus penetration.

### Oil-in-water adjuvants increase virus uptake and enhance antigen expression

**Fig. 8.**
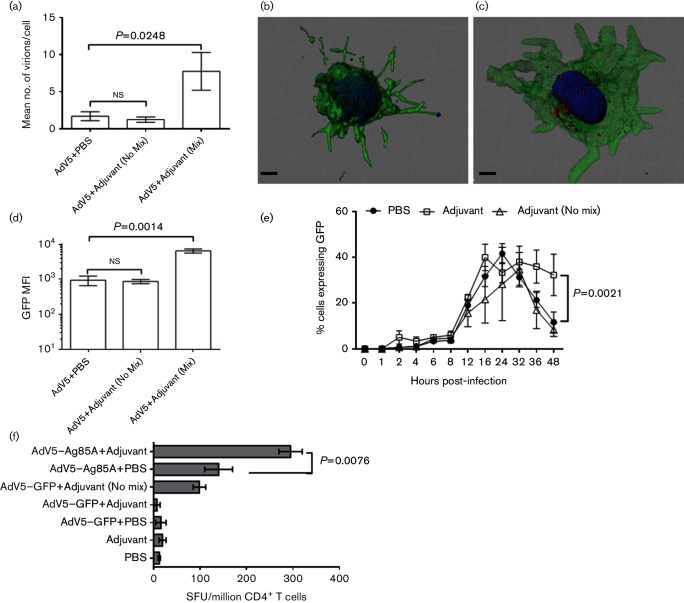
Oil-in-water adjuvants improve AdV5 uptake by ALDC, increase and prolong recombinant antigen expression and enhance antigen presentation. AF568-labelled AdV5 was mixed with PBS or an oil-in-water adjuvant, added to ALDC, cultured overnight and washed twice prior to analysis. (a) Confocal microscopy was used to count the number of intracellular virions per cell 4 h post-infection. (b and c) Three-dimensional reconstructions of representative samples of ALDC infected with AdV5-568 in PBS or adjuvant respectively 4 h post-infection. (d) GFP MFI of ALDC infected with AdV5-GFP in PBS or adjuvant 24 h post-infection. (e) Frequency of GFP-expressing ALDC over time following infection with AdV5-GFP in PBS or adjuvant. (f) Ag85A-specific IFN-γ ELISpot using AdV5-Ag85A or AdV5-GFP in PBS or adjuvant. ALDC were infected with AdV5 recombinants as described in Methods and cultured with MHC-matched CD4^+^ T cells from BCG-vaccinated animals. In all cases, the bars indicate means of 20 imaged cells from three different animals analysed in duplicate and error bars indicate standard error of the means.

Various approaches have been proposed to improve targeting of AdV vectors to DC, including modification of the virus fibre protein to increase virus binding and penetration to DC. Previous observations by us and others (Cubillos-Zapata *et al.*, 2011; Ganne *et al.*, 1994) indicate that the use of oil-in-water emulsions as adjuvants to deliver AdV5-based vaccines significantly increases the magnitude of immune responses to the trans-gene *in vivo*. To identify the effect of adjuvanted vector on ALDC, we prepared AF568-labelled AdV5-GFP in an oil-in-water emulsion or combined it with the adjuvant without mixing, and we incubated ALDC with the preparations. ALDC incubated with emulsified virus contained a significantly greater number of intracellular virions compared to cells incubated with virus in adjuvant without mixing or in PBS (*P*=0.0248; Fig. 8a–c). The MFI of GFP was greater (Fig. 8d) and expression was more sustained (Fig. 8e) in ALDC incubated with emulsified virus than in cells incubated with the virus in adjuvant without mixing or in PBS. However, the percentage of cells expressing the trans-gene did not change (Fig. 8e). To confirm the effect of adjuvanted vector on antigen presentation, we prepared AdV5 expressing mycobacterial antigen 85A [Ag85A (Cubillos-Zapata *et al.*, 2011)] in adjuvant with or without mixing and incubated it with ALDC. These ALDC were then cultured with CD4^+^ T cells obtained from MHC-matched, BCG-vaccinated cattle. Ag85A-specific responses were significantly higher when AdV5-Ag85A was mixed with adjuvant compared to AdV5-Ag85A without mixing or without adjuvant (*P*=0.0076). The number of IFN-γ producing cells was minimal when T cells were cultured with ALDC exposed to AdV5-GFP with or without adjuvant or to adjuvant alone (Fig. 8f). These data show that, in the absence of genetic modification of the virus fibre, increased transduction efficiencies can be achieved by the use of water-in-oil adjuvants.

## Discussion

We have previously shown that, in contrast to monocyte-derived DC, ALDC can be readily transduced by replication-deficient AdV5 (Cubillos-Zapata *et al.*, 2011), achieving up to 50 % transduction of ALDC *in vitro* and up to 12 % *in vivo*. ALDC can be separated into two main subpopulations, CD172α^+^ and CD172α^−^; only the former can be transduced by AdV5 vectors both *in vitro* and *in vivo* (Cubillos-Zapata *et al.*, 2011). In the current study, we aimed to characterize the mechanism of AdV5 uptake by ALDC further.

Since its discovery as a primary receptor for AdV5 (Bergelson *et al.*, 1997), it has been widely accepted that CAR is involved in AdV5 attachment to target cells. However, a number of other cell surface molecules, such as CD40, MHCII, CD46 and Fc receptors, have been identified, which appear to play an important role in AdV5 attachment [reviewed in Zhang & Bergelson (2005)]. We could not prevent the transduction of ALDC by AdV5 following proteolytic cleavage of surface proteins or by using RGD blocking peptides. Although there is evidence that DC-SIGN is involved in attachment of AdV5 to human monocyte-derived DC (Adams *et al.*, 2009), we could not replicate these results using bovine ALDC. This may be due to differences in the interaction of AdV5 between human and bovine DC or to differences between monocyte-derived DC and ALDC. Apart from an antibody against CD64 (FcγR), antibodies against bovine FcγR are not available; thus, it is possible for these receptors to be involved in AdV5 entry into bovine ALDC. VOPBA has been used to identify the binding of dengue virus (Jindadamrongwech & Smith, 2004), pseudorabies virus (Karger & Mettenleiter, 1996) and respiratory syncytial virus (Tayyari *et al.*, 2011) to cell receptors. We used this assay under denaturing and non-denaturing conditions to determine whether a protein receptor for AdV5 on membrane-enriched fractions from ALDC could be identified. Although binding of virus to a protein present in enriched membrane fractions from 293 and BL cells could be detected, we could not identify binding of AdV5 to membrane proteins derived from ALDC (Fig. 1). It is possible that our inability to identify an AdV5 receptor on ALDC is due to the stringency of the assay’s conditions or the sensitivity of our assays. New technologies developed to identify protein–protein interaction, such as fluorescence resonance energy transfer or high-affinity co-immunoprecipitation followed by highly sensitive mass spectrometry, may help identify receptors involved in AdV attachment in the future.

Using biotinylated AdV5, we showed that the virus does not bind to the surface of ALDC as it does to the surface of 293 cells, suggesting that internalization of AdV5 by bovine ALDC is mediated by CAR-independent macropinocytosis, and similar pathways have been described for internalization other viruses such as influenza (de Vries *et al.*, 2011) and vaccinia (Sandgren *et al.*, 2010) by DC. Alternatively, it is possible for AdV5 to use low-affinity receptors present on the surface of ALDC as observed in other systems, such as CD46 (Sirena *et al.*, 2004), αMβ2 and αLβ2 (Huang *et al.*, 1996) integrins. It is possible that labelling the virus with sulfo-NHS conjugates (biotin, AF569 or fluorescein) changes the way the virus enters the cells. We have tried to address this possibility by using non-labelled virus as control and GFP as readout of transduction when at all possible.

Professional phagocytic cells such as DC have the capacity to take up small and large particles using a variety of mechanisms such as macropinocytosis and micropinocytosis (Platt *et al.*, 2010; Savina & Amigorena, 2007). Using biochemical inhibitors of endocytosis, we found that cytochalasin D was able to block AdV5 uptake indicating that AdV5 internalization by ALDC is an actin-mediated process (Cooper, 1987) and that skin-migrating DC utilize their capacity as professional phagocytic cells to survey peripheral sites acquiring foreign antigens such as vaccine vectors, as well as processing these antigens prior to and on arrival to local draining lymph nodes. Interestingly, dynasore had no effect on endocytosis of AdV5 indicating that this process is clathrin independent (Chen *et al.*, 2009) and therefore does not require the binding of RGD to cellular integrins. This suggestion is supported by the finding that RGD blocking peptides did not inhibit AdV5 internalization by bovine ALDC (Fig. 2).

We then looked at events following virus internalization and, using confocal microscopy, we observed occasional co-localization of AdV5 particles with the early endosome marker EEA1, but not with the late endosome marker LAMP1 (data not shown), supporting previous evidence of AdV5 exit from early endosomes to the cytosol (Svensson & Persson, 1984). Although it has been previously proposed that AdV5 exits early endosomal compartments following intra-endosome acidification [see Greber *et al.* (1993) and reviewed in Smith *et al.* (2010)], we could not block virus transduction of ALDC using a number of lysosomotropic agents. Additionally, the fluorescence intensity of AdV5-fluorescein remained constant in ALDC over time whereas the fluorescence intensity of fluorescein-labelled dextran declined as fluorescein became protonated (Fig. 7b and d). Our data indicate that acidification of endosomes is not required for transduction of ALDC by AdV5, and this has been shown to be the case in other systems (Otero & Carrasco, 1987; Rodriguez & Everitt, 1996; Svensson & Persson, 1984), and although Suomalainen *et al*. found in epithelial cells that virus penetration is independent of low endosomal pH, it could still be inhibited by ammonium chloride; thus, it is possible that AdV5 exit to the cytosol requires acidification of endosomes in some cells but not others. This raises the question of how AdV5 exits endosomal compartments in ALDC. It has been proposed that viral (Suomalainen *et al.*, 2013) or cellular proteases degrade early endosomes (Wiethoff *et al.*, 2005), or perhaps a yet unknown mechanism is involved in this process and requires further investigation.

The presence of naturally acquired neutralizing antibodies against AdV5 is one of the major obstacles in the deployment of effective recombinant AdV5 vaccine vectors (Ahi *et al.*, 2011). Antibodies bound to neutralizing epitopes on the virus surface normally prevent virus binding to the cell’s receptors (Roy *et al.*, 2005; Sumida *et al.*, 2005). However, we observed that AdV hyperimmune bovine sera enhanced the uptake of AdV5 by ALDC, perhaps through the use of Fc receptors while blocking trans-gene expression. We could not test the effect of blocking Fc receptors due to the lack of available reagents to use in bovine cells. In the presence of AdV5-specific antibody, the AF568-labelled virions were not translocated to the nuclear membrane but remained in the cytoplasm and the signal was eventually lost (data not shown). A similar phenomenon has been described previously in HeLa cells (Smith *et al.*, 2008), in which TRIM21 binds to antibody–AdV5 complexes and targets the complexes for proteasomal degradation (Mallery *et al.*, 2010). The role of TRIM21 in AdV5 degradation remains to be investigated in DC.

Various approaches have been proposed to improve targeting of AdV vectors to DC, including modification of the virus fibre protein to increase virus binding and penetration to DC. In light of our current results and previous studies that have shown that AdV5 emulsified in an oil-in-water adjuvant and injected over the site of cannulation provides longer trans-gene expression and improved immunogenicity compared to non-adjuvanted virus (Cubillos-Zapata *et al.*, 2011), we sought to understand the mechanism of improved immunogenicity in the absence of clear virus fibre–receptor interactions. Cells transduced with adjuvanted virus contained more intracellular virions and trans-gene expression was stronger and longer lasting than in cells transduced with virus in the presence of adjuvant but without emulsification, or in PBS. In presentation assays, antigen-specific IFN-γ T-cell responses were higher when adjuvanted virus was used. This suggests that the oil-in-water emulsion provides a biological medium which is used by the DC to take up larger amounts of solute; in this case, AdV5. This in turn translates into greater numbers of intracellular virions that have the capacity to translocate to the nucleus more effectively and for longer periods of time, or that the virions may be protected from intracellular degradation for a longer period of time when taken up in an adjuvant emulsion. This results in stronger trans-gene expression and thus antigen presentation. In addition, the adjuvant emulsion may activate TLRs which provide signals for the DC to be more effective at activating T cells. However, our controls suggest that this may not be the case since *in vitro* responses to AdV5-Ag85 in PBS are not significantly higher than responses to AdV5-Ag85 in adjuvant but without mixing (Fig. 8f). Further studies are required to understand the relationship between biochemical adjuvants and DC. Ultimately, genetic modification of fibre protein will only be useful if a clear cellular receptor is identified in the target cell; therefore, alternative approaches, such as oil-in-water emulsions, may be the most appropriate to improve AdV-based gene delivery.

In conclusion, here we describe the interaction of a replication-deficient AdV vector with skin-migrating bovine DC, which are collected by cannulation of lymphatic vessels and are not subject to culture under laboratory conditions. We present evidence of the phagocytic action of these DC. Upon encountering virus, ALDC actively phagocytose the virus particles, perhaps using an unknown low-affinity receptor and which takes between 3 and 4 h before virus particles can be observed intracellularly. Following entry, the virus quickly exits endosomal compartments via an unknown mechanism or is never associated with acidic endosomes, travelling to the nuclear membrane and thus initiating trans-gene transcription and translation. Neutralizing antibodies not only prevent virus entry into DC but enhance it while inhibiting translocation to the nucleus. Our data will be useful in understanding DC–vaccine interactions and will help further development and improvement of viral vectors. Defining and manipulating entry pathways may enhance vaccine vector efficacy through improved antigen presentation.

## Methods

### Pseudoafferent lymphatic cannulation.

MHC-defined (Ellis *et al.*, 1996, 1998), conventionally reared, 6-month-old Friesian Holstein calves (*Bos taurus*) from The Pirbright Institute (Pirbright) herd were used for these studies. Cannulations were performed essentially as described previously (Hope *et al.*, 2006). Lymph was collected into sterile plastic bottles containing heparin (10 U ml^−1^), penicillin (100 U ml^−1^) and streptomycin (100 µg ml^−1^). The lymph collected was either used fresh or centrifuged (300 ***g***, 8 min) and resuspended in heat-inactivated foetal calf serum (FCS; Autogen Bioclear) containing 10 % DMSO, and the cells were stored in liquid nitrogen prior to use. Mononuclear cells were isolated from afferent lymph by density gradient centrifugation over Histopaque 1083 (Sigma). *Mycobacterium bovis* Ag85A-specific T cells were obtained from MHC-defined cattle vaccinated subcutaneously with 10^6^ c.f.u. of BCG Pasteur. All T cells used were collected 3 weeks post-vaccination at the peak of the response. All animal experiments were approved by the Pirbright’s ethics committee and carried out according to the UK Animal (Scientific Procedures) Act 1986.

### Cell lines and primary cells.

HeLa cells and 293 were obtained and maintained by the Microbiological Services Department (Pirbright) in tissue culture media in the absence of antibiotics. CHO cells expressing human recombinant CAR were provided by Dr M. Cottingham, Jenner Institute, University of Oxford, UK. Bovine ALDC (FSC^high^ MHCII^+^ DEC205^+^ CD11c^+^ CD8α^−^) were separated from other lymph-migrating cells using a FACSAria II (Becton Dickinson) and purities were confirmed by flow cytometry using FACSDiva v6 (Becton Dickinson). Peripheral blood CD14^+^ monocytes, CD4^+^ and CD8^+^ T cells were magnetically separated using anti-human CD14 (Miltenyi Biotech), CC30 and CC63 monoclonal antibodies (Guzman *et al.*, 2008), respectively, and MACS technology (Miltenyi Biotech) following the manufacturer’s instructions. Typically, the purity of the resulting dendritic and T-cell subsets was over 97 % as determined by flow cytometry as described above.

BL cells were isolated from Holstein cattle at the time of slaughter and were provided by Pirbright’s Microbiological Services Department (Chanter *et al.*, 1986).

### Monoclonal antibodies and flow cytometry.

Fluorochrome-labelled mouse anti-bovine monoclonal antibodies used in this study have been described in detail previously (Brooke *et al.*, 1998; Howard *et al.*, 1991, 1997; Howard & Naessens, 1993; Sallusto *et al.*, 1995). These were CC98-APC (anti-DEC205), CC14-PE (anti-CD1b), CC149-PerCP/Cy5.5 (anti-SIRPα), IL-A16-AlexaFluor 680/PE (anti-CD11c), IL-A21-PE (anti-MHCII), IL-A88-FITC (anti-MHCI), IL-A156-PE (anti-CD40), N32/52-3-PE (anti-CD80) IL-A159-PE (anti-CD86), CC30-APC/Cy5.5 (anti-CD4), CC63-APC/Cy7 (anti-CD8), IL-A111-AlexaFluor 610/PE (anti-CD25) and CC302-PE (anti-IFN-γ). Isotype- and concentration-matched anti-turkey rhinotracheitis virus monoclonal antibodies were used as controls (Hope *et al.*, 2006; Whelan *et al.*, 2003). Dead cells were excluded using the 405 nm excitable dye Live/Dead Aqua or propidium iodide (Invitrogen) following the manufacturer’s instructions. The cells were acquired using an LSRFortessa (Becton Dickinson) and staining was analysed using FCS Express v4 (DeNovo Software). ALDC were distinguished from other cells on the basis of their high forward scatter (FSC), expression of MHCII, CD11c and high-intensity expression of DEC205 and lack of CD8α (Gliddon *et al.*, 2004; Hope *et al.*, 2006). Only live, single events were used for analysis.

### Viruses.

E1- and E3-deleted recombinant AdV5 expressing GFP or mycobacterial antigen 85A (Ag85A) was generated by the Jenner Institute Viral Vector Core Facility, University of Oxford, UK, as described previously (Cubillos-Zapata *et al.*, 2011).

For some assays, aliquots of 1×10^11^ virus particles of AdV5 were labelled with NHS-AlexaFluor568, NHS-biotin or NHS-fluorescein (Invitrogen) following the manufacturer’s instructions and labelled virus was dialysed twice against PBS. The virus was then titrated in 293 cells and GFP expression was measured by flow cytometry. In some cases, the titre was found to be 1 log lower after labelling and the infectious doses were adjusted as required.

### Generation of bovine hyperimmune sera to AdV5.

Three 6-month-old Holstein-Friesian calves were inoculated intramuscularly with 1×10^9^ virus particles of purified AdV5 three times at 6-week intervals. The hyperimmune sera used were collected 6 weeks after the last immunization, pooled and tested in a virus neutralization assay (Sumida *et al.*, 2005).

### Infection of afferent lymph cells.

Migrating cells from the afferent lymph were cultured [IMDM containing 10 % FCS and 10^−5^^ ^M 2-β-mercaptoethanol (Sigma-Aldrich)] with the recombinant viruses using optimal MOI values, as described previously (Cubillos-Zapata *et al.*, 2011). In some assays, transferrin-AF568 (Sigma) and dextran-fluorescein (Invitrogen) were used as tracing markers. In neutralization assays, AdV5 was incubated with 1 µg of anti-hexon antibody raised in goats (Millipore) or with bovine pre-immune or hyperimmune sera raised against human AdV5 generated by immunizing calves with 1×10^9^ virus particles of AdV5 as described above.

### Detection of known adenovirus receptors and removal from cell surface.

To analyse the expression of known adenovirus receptors on ALDC and BL cells, we used the following antibodies in flow cytometry: N-17 (goat anti-CAR, Santa Cruz Biotechnology), N32/52-3 (anti-CD80), IL-A159 (anti-CD86), IL-A88 (anti-MHCI), IL-A21 (anti-MHCII), T320.11 (anti-heparin/heparin sulphate, Merck), 2-2B (anti-sialic acid, Merck), CC30 (anti-CD4), CC63 (anti-CD8), 344519 (anti-CD46, R&D Systems), CCG24 (anti-FcγRII), KD1 (anti-FcγRIII, Abcam), C-20 (anti-DC-SIGN, Santa Cruz Biotechnology), LM609 (anti-a_v_β_3_, Chemicon), monoclonal antibody 2000 (anti-a_v_β_1_, Chemicon) and 10D5 (anti-a_v_β_6_, Millipore). All antibodies were obtained from Pirbright except where noted. Antibodies were added to cells (1 µg 10^−6^ cells) and incubated at 4 °C for 60 min. After three washes with PBS, the cells were stained with AF647-labelled goat-, rabbit- or mouse-specific secondary antibodies (Serotec) and the cells were analysed by flow cytometry as described above.

Digestion of cell surface proteins was achieved using a mixture of proteolytic enzymes (Wald *et al.*, 2001) consisting of 2 U of trypsin, 1 U of papain and 2 U of chymotrypsin (Merck). We treated 10^6^ cells with the enzyme mix in a volume of 100 µl for 30 min at 37 °C; the cells were then washed twice in cold PBS and resuspended in culture media containing a final concentration of 5 µg ml^−1^ of brefeldin A (Sigma).

### Virus attachment assay by ELISA.

BL cells, ALDC, CD14^+^ monocytes or 293 cells were cultured on 96-well plates. Antibodies against known AdV5 receptors were added to the cells at 1 µg 10^−6^ cells; in some cases, the following chemical agents known to block AdV5 entry were also added: 10 U of sodium heparin (Sigma), 1 U of trypsin (Sigma) or 10 mM RGD antagonist Cyclo(Ala-Arg-Gly-Asp-3-Aminomethylbenzoyl) (Sigma). After 1 h of incubation at 37 °C, the cells were washed in cold PBS, and biotinylated AdV5 (MOI=100 virus particles per cell) was added for 90 min on ice or at 37 °C. The cells were then washed three times with ice-cold PBS and fixed with 3 % paraformaldehyde. After blocking with 1 % BSA in PBS, streptavidin-HRP (Sigma, 1 : 500) was added and the plates were incubated for 60 min at room temperature. The plates were washed with PBS-Tween and the plates were developed with TMB Turbo (Pierce). Reactions were stopped with 1 M H_2_SO_4_ and optical densities were measured using a FluorostarOptima (BMG Labtech).

### VOPBA and Western blot.

VOPBA was carried out essentially as described by Cao *et al.* (1998) with a few modifications. Subcellular fractions from 1×10^6^ 293, BL and ALDC were enriched using the ProteoExtract subcellular fractionation kit (Merk Millipore) following the manufacturer’s instructions. Total cell protein and membrane fractions were separated by PAGE on 4–10 % denaturing and non-denaturing TGX stain-free gels (Bio-Rad) and transferred onto Immun-Blot PVDF membranes (Bio-Rad). The membranes were blocked with 5 % (w/v) dry milk-PBS overnight, rinsed with PBS and probed with AdV5 (1×10^8^ virus particles in 10 ml of milk-PBS) for 90 min. The membranes were then washed three times with PBS and incubated with 10 µg of biotinylated goat anti-AdV5 (Serotec) in 10 ml of milk-PBS for 60 min. The membranes were washed three times and incubated with 10 µg of streptavidin-conjugated HRP (Dako) in 10 ml of milk-PBS for 60 min. After extensive washing, we developed the membranes with Immun-Star WesternC substrate (Bio-Rad) and visualized them using a ChemiDocMP digital imager (Bio-Rad). For competition VOPBA and before the addition of AdV5, 10 µg of rabbit anti-CAR pAb (Abcam) was added to the membranes and incubated for 90 min at room temperature. After washing three times with PBS, we probed the membranes with AdV5 and the assay was carried out as described above.

For detection of CAR by Western blot, membrane fractions separated by PAGE were transferred onto PVDF membranes, blocked with milk-PBS and probed with 10 µg of rabbit anti-CAR pAb or 1 µg of anti-β actin mouse monoclonal antibody (Abcam) in 10 ml of milk-PBS. After washing with PBS containing 1 % Tween 20 (PBS-T), we incubated the membranes with 1 µg of anti-goat or anti-mouse antibody conjugated to HRP (Dako) in 10 ml of milk-PBS for 60 min. After extensive washing with PBS-T, we developed the membranes as described above.

### Biochemical inhibitors.

The following inhibitors and final concentrations were used to block endocytosis: cytochalasin D [1 µM, actin-dependent (Sakr *et al.*, 2001)]; filipin [5 µg ml^−1^ caveolae-dependent (Rothberg *et al.*, 1992)]; chlorpromazine [10 µg ml^−1^, prevents clathrin-coated pit formation (Wang *et al.*, 1993)]; methyl-β-cyclodextrin [10 mM, cholesterol-dependent (Vieth *et al.*, 2010)]; amiloride [1 mM, Na^+^ blocker (West *et al.*, 1989) and therefore blocks macropinocytosis (Sallusto *et al.*, 1995)]; ciliobrevin [10 µM, inhibitor of motor cytoplasmatic dynein (Firestone *et al.*, 2012); and dynasore [8 mM, inhibitor of dynamin- and clathrin-dependent endocytosis (Macia *et al.*, 2006)].

The following lysosomotropic agents were used to block acidification of endosomal compartments: bafilomycin [1 µM, inhibitor of vacuolar-type H^+^-ATPase (Yoshimori *et al.*, 1991)]; NH_4_Cl [a weak base (Sonawane *et al.*, 2002) and diluted in PBS]; and chloroquine [10 µM, inhibits endosomal maturation (Mellman *et al.*, 1986)].

All inhibitors were diluted in DMSO, except where noted. DMSO was used as diluent control and PBS was used as a negative control. All chemicals were obtained from Sigma-Aldrich. Cells (1×10^6^ final) were plated in triplicate in culture media in 96 U-bottomed plates, and the biochemical inhibitors were added to the final concentrations described above in a final volume of 100 µl and mixed thoroughly. After 1 h of incubation at 37 °C, virus internalization assays were performed as described below.

### Virus internalization assay.

To differentiate between attachment and entry, we modified a fluorometry phagocytosis assay (Wan *et al.*, 1993) based on the capacity of trypan blue to quench extracellular fluorescein. AdV5 was labelled with NHS-fluorescein (Pierce) following the manufacturer’s instructions. ALDC or 293 cells were cultured in 96-well plates with the labelled virus (MOI=100 virus particles per cell) for 6 h or 60 min, respectively, at 37 °C or at 4 °C in the presence of the biochemical inhibitors described above. All inhibitors prepared in DMSO were diluted in culture media and 10 % DMSO in culture media was used as negative control. After the required incubation period, trypan blue (0.5 % final; Sigma) and Live/Dead Aqua (Invitrogen) were added and the cells were analysed by flow cytometry; 25 000 live/single events were used to generate statistical analyses.

### Confocal microscopy.

FACS purified DC (FSC^high^ MHCII^+^ DEC205^+^ CD11c^+^ CD8α^−^) were cultured on collagen-treated coverslips (Sigma) in the presence (MOI=10) or absence of labelled AdV5. The cells were fixed with 3 % paraformaldehyde for 20 min, washed twice with PBS and permeabilized with 0.1 % Triton X-100 in PBS. Anti-EEA1 polyclonal antibody raised in rabbits (1 µg ml^−1^ final concentration; Abcam) was used to visualize early endosomal compartments; the tracer molecules dextran and albumin (both at 0.5 µg ml^−1^ final concentration) conjugated to AF488 were used for co-localization assays; rabbit anti-FMDV was used as a negative control (final dilution of 1 : 750). Goat anti-rabbit-AF488 (1 µg ml^−1^; Invitrogen) was used as secondary antibody and all samples were counterstained with DAPI (100 nM; Invitrogen) following the manufacturer’s instructions. Where indicated, Phalloidin-AF488 (0.2 U per slide; Invitrogen) was used to identify actin filaments. Cells were mounted onto microscope slides using VectaShield (Vector Laboratories) and observed using a 65× lens mounted on a Leica SP5 confocal microscope. The Leica LAS AF software was used to take sequential, three-dimensional stack images in the Z-plane acquiring stacks of 80–120 optical sections from infected cells as optimized by the software. The number of intracellular virions, three-dimensional images and co-localization data sets were analysed using Bitplane Imaris 6.4.2 image analysis software (Bitplane) with surface smoothing of 0.05 nm for nuclei (blue) and 0.02 nm for AdV (red) and endosomes (green).

### pH-dependent fluorometry.

Dextran-fluorescein (Invitrogen) was used to measure intracellular pH essentially as described previously (Downey *et al.*, 1999). Calibration of the fluorescence ratio versus pH was performed for each experiment by equilibrating the cells in isotonic K^+^-rich medium buffered to varying pH values (between 5.0 and 7.5) in the presence of the K^+^/H^+^ ionophore nigericin (5 mM; Sigma). Calibration curves were constructed by plotting the extracellular pH, which was assumed to be identical with the cytosolic pH under these conditions, against the corresponding fluorescence ratio. AdV5 (MOI=100) or dextran (25 µg ml^−1^) labelled with fluorescein was added to ALDC or 293 cells cultured in triplicate in 96-well plates (Costar). Real time fluorometry was measured every 30 min using an Infinite M200 (Tecan) and the results were analysed using Magellan for Windows (Tecan).

### Generation of oil-in-water emulsions.

AdV5 recombinants expressing GFP or Ag85A were mixed with the adjuvant Montanide ISA 206V (SEPPIC) to form oil-in-water emulsions following the manufacturer’s instructions. Briefly, 1×10^9^ virus particles in a volume of 250 µl were mixed with an equal volume of adjuvant and vigorously mixed for 2 min. Two negative controls were also prepared, one containing AdV5 and adjuvant but without mixing and the other one with PBS mixed with ISA 206V and emulsified as described above. The emulsions containing 100 virus particles per cell were added to 96-well U-bottomed tissue culture plates containing 1×10^5^ ALDC in 100 µl of media and mixed by pipetting until the solution looked homogenous. In the case of the control containing AdV5 and adjuvant without mixing, the solutions were mixed only once and clear hydrophobic/hydrophilic globules observed macroscopically. The infection was allowed to continue as described in the text and was washed twice with PBS before analysis.

### Antigen presentation assays.

Ag85A-specific IFN-γ producing lymphocytes were analysed using ELISpot assays as described previously (Guzman *et al.*, 2012; Hope *et al.*, 2012) utilizing purified CD4^+^ T cells from MHC-matched, BCG-vaccinated animals (Thom *et al.*, 2012).

### Statistical analysis.

Calculation of descriptive statistics (geometric statistics, standard error of the means and standard deviations), two-way parametric ANOVA including multiple comparisons, Bonferroni multiple comparison tests and graphs were generated using GraphPad Prism for Windows v6.01 (GraphPad).

## Supplementary Data

142 Supplementary File 1
