# Supplementary material for: Transduction of skin-migrating dendritic cells by human adenovirus 5 occurs via an actin-dependent phagocytic pathway
Source: J Gen Virol. 2016 Oct 13;97(10):2703–18. doi: 10.1099/jgv.0.000581 (PMC5078831; doi:10.1099/jgv.0.000581)
Supplement: Supplementary File 1 [file jgv-97-2703-s001.docx]

Supplementary Figure 1


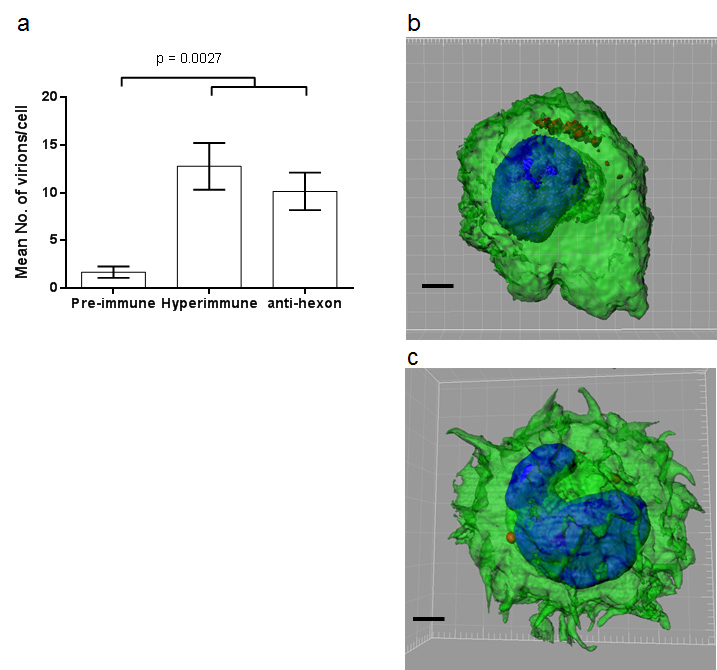


**S1.** Neutralizing antibodies do not prevent virus entry into ALDC. AF568-labelled AdV5 was mixed with PBS, normal bovine sera or bovine hyperimmune sera to AdV5 and incubated for 60 min after which the mix was added to ALDC as described in Materials and Methods and confocal microscopy was used to quantify the number of intracellular AF568-labelled virions. a) Mean number of intracellular AF568^+^ particles per cell. Bars indicate means of cells from 5 different animals analysed in duplicate and error bars indicate standard error of the means. b and d) Three-dimensional reconstructions from confocal micrographs showing ALDC cultured for 5 hrs with AdV5-AF568 previously incubated with hyperimmune sera (b) or normal sera (c). Reconstructions are representative of cells from 5 independent experiments. Black bar indicates 5 microns.
